# Induced Systemic Resistance for Improving Plant Immunity by Beneficial Microbes

**DOI:** 10.3390/plants11030386

**Published:** 2022-01-30

**Authors:** Yiyang Yu, Ying Gui, Zijie Li, Chunhao Jiang, Jianhua Guo, Dongdong Niu

**Affiliations:** 1 College of Plant Protection, Nanjing Agricultural University, Nanjing 210095, China; yyy@njau.edu.cn (Y.Y.); 2020202009@stu.njau.edu.cn (Y.G.); 2019202003@njau.edu.cn (Z.L.); chjiang@njau.edu.cn (C.J.); jhguo@njau.edu.cn (J.G.); 2 State Key Laboratory of Biological Interactions and Crop Health, Nanjing Agricultural University, Nanjing 210095, China; 3 Engineering Center of Bioresource Pesticide in Jiangsu Province, Nanjing 210095, China

**Keywords:** induced systemic resistance, beneficial microorganism, defense response, small RNA

## Abstract

Plant beneficial microorganisms improve the health and growth of the associated plants. Application of beneficial microbes triggers an enhanced resistance state, also termed as induced systemic resistance (ISR), in the host, against a broad range of pathogens. Upon the activation of ISR, plants employ long-distance systemic signaling to provide protection for distal tissue, inducing rapid and strong immune responses against pathogens invasions. The transmission of ISR signaling was commonly regarded to be a jasmonic acid- and ethylene-dependent, but salicylic acid-independent, transmission. However, in the last decade, the involvement of both salicylic acid and jasmonic acid/ethylene signaling pathways and the regulatory roles of small RNA in ISR has been updated. In this review, the plant early recognition, responsive reactions, and the related signaling transduction during the process of the plant–beneficial microbe interaction was discussed, with reflection on the crucial regulatory role of small RNAs in the beneficial microbe-mediated ISR.

## 1. Introduction

With the rapid growth of the world’s population, people’s demand for agricultural products is increasing. Plants are sessile organisms, frequently exposed to a myriad of microorganisms, including pathogenic and beneficial ones. The pursuit of productivity has led to the abuse of fertilizers and pesticides, causing serious environmental pollution and ecological damage. During development, the main concerns in the agricultural industry have changed from yield to food quality and environmental impact. The use of environmentally friendly agricultural inputs has arisen since then. Biological control uses beneficial organisms to suppress harmful organisms and promote plant growth. Currently, many beneficial microorganisms, such as *Bacillus*, *Pseudomonas*, and *Trichoderma*, are used as biological control agents to control field plant diseases.

Plants possess an innate ability to sense and recognize potential invading microorganisms and to activate defense responses [1]. On the contrary, to perceive the beneficial microorganisms and form a symbiotic relationship with them, plants adopt similar, yet distinct, cell surface receptors [2]. Plants can recognize microbial- or pathogen-associated molecular patterns (MAMPs or PAMPs), such as bacterial flagellin and fungal chitin, through transmembrane pattern recognition receptors (PRRs), and this process triggers the first layer of immune defense, named pattern-triggered immunity (PTI) [3]. However, pathogens can overcome the first layer by suppressing PTI signaling or evading recognition of PRRs by secreting virulence effectors [4]. Effectors are a kind of virulence-associated molecule, delivered by pathogens via microbial secretion systems into plant cells or the apoplast to suppress host immunity [4]. In turn, the second layer of plant immunity, called effector-triggered immunity (ETI), evolved to recognize pathogen effectors through polymorphic NB-LRR proteins (possessing nucleotide-binding and leucine-rich repeat domains), resulting in hypersensitive reaction (HR) to limit the pathogen spread [5]. Interestingly, recent studies showed that PRRs are also required for ETI [6]. The complex and precise immune system built from host–pathogen competition allows beneficial microorganisms to induce plant immunity through targeting the key elements in the process of PTI and ETI by modulating host small RNAs.

Plant systemic resistance can be divided into induced systemic resistance (ISR) and systemic acquired resistance (SAR), induced by non-pathogenic microbes and pathogenic microbes, respectively [7,8]. Colonization by beneficial microbes induces a physiological state of plant host called “priming”. Upon the activation of “priming”, plants display stronger and faster defense responses against the following invasion of pathogens, demonstrated as a common feature of systemic resistance induced by beneficial microorganisms [9]. SAR was first discovered in 1961 and identified as a salicylic acid (SA)-dependent plant defense, featured by accumulation of SA and activation expression of pathogenic-related (PR) genes [10,11]. In 1991, three research groups independently and specifically evidenced that beneficial microbes enhanced plant immunity by ISR [12,13,14]. Among these three groups, Kloepper et al. found that plant growth-promoting rhizobacteria (PGPR) could induce cucumber systemic resistance to *Fusarium*-wilt, bacterial angular leaf spot, root-knot nematode, and cucumber mosaic cucumovirus [13,15,16,17,18]. In 1996, Pieterse et al. firstly reported that systemic resistance induced by PGPR was independent of SA and PR proteins in *Arabidopsis thaliana*, but depended on jasmonic acid (JA) and ethylene (ET) pathway [19,20], which was proposed to be the difference between ISR and SAR. Nevertheless, multiple following reports demonstrating activation of both SA and JA/ET signaling pathways in ISR triggered by beneficial microbes revealed the complexity and diversity of signal pathways involved in ISR [21,22,23,24].

Up to now, various beneficial microorganisms have shown the potential to induce systemic resistance. Beneficial bacteria, such as *Bacillus* spp. and *Pseudomonas* spp., can stimulate defense responses and help plants to obtain broad-spectrum disease resistance [14,25]. Beneficial fungi, such as *Trichoderma* spp. and arbuscular mycorrhizal fungi (AMFs), have been considered to be widespread potential biocontrol agents [26,27]. Root treatment with *Trichoderma harzianum* T39 induced ISR in bean against *Botrytis cinerea* [27]. AMFs, which form symbiotic associations with many plant root systems, have been proved to induce local and systemic resistance to *Phytophthora parasitica* in tomato roots [26].

In this review, we summarize the recognition of beneficial microorganisms and early events that occur during induced systemic resistance, highlighting reactive oxygen species burst, callose deposition that can inhibit the infection and expansion of pathogens, calcium signaling, and transcriptional factors, that play a significant role in regulating the expression of downstream defense-related genes and diseases control. The crosstalk of signaling transduction pathways and the function of secondary metabolites and stomatal regulation in ISR will be discussed. Finally, we will highlight recent advances about the role of small RNAs in rhizobacteria-induced ISR.

## 2. Recognition of Beneficial Microbes by Plants

Plants can sense the beneficial microbes by recognizing the common microbial compounds they produce, such as flagellin, lipopolysaccharides (LPS), exopolysaccharides, and chitin oligosaccharides, such as ligands [2,28,29,30]. Binding with ligands, the receptor proteins recruit co-receptors and form complexes to phosphorylate downstream substrates, leading a signal cascade involving oxidative burst, Ca^2+^ influx, MAPK activation, and hormone signaling activation [31].

The N-terminal part of flagellin, including the 22-amino acid epitope flg22, is highly conserved in a wide range of eubacteria [32]. The flagellin from beneficial microbes, such as *Bacillus subtilis* and *Burkholderia phytofirmans*, can be recognized by their host plants [32,33]. FLAGELLIN-SENSING 2 (FLS2) is the first reported receptor to recognize flagellin from PGPR [34]. The perception of flg22 results in the heterodimerization between FLS2 and its co-receptors, BRI1-associated kinase (BAK1) and BAK1-LIKE1 (BKK1), which phosphorylate their interacting receptor-like cytoplasmic kinase *Botrytis*-induced kinase1 (BIK1) to initiate the PTI signaling [35,36,37]. *Arabidopsis thaliana bak1* mutations showed normal flagellin binding but abnormal PTI responses, indicating that BAK1 acted as a positive regulator in signaling [36]. BIK1 is phosphorylated upon flagellin perception and subsequently, transphosphorylates FLS2/BAK1 complex to transmit flagellin signaling and activate intracellular signaling cascades [37]. Similar to *bak1* mutant, *bik1* mutant is compromised in flagellin-mediated responses to the invasion of non-pathogenic microbes, indicating that BIK1 is an essential component in MAMP signal transduction and induced systemic resistance. In addition, rhizobia and AMF establish symbiosis with the host by means of chitin-derived oligosaccharides signals [2]. Nod factors, for instance, are acylated lipo-chitooligosaccharides, delivered by rhizobia and recognized by LysM receptor-like kinases to activate a common symbiotic pathway, which controls both the arbuscular mycorrhizal symbioses and the rhizobia-legume to form mycorrhization and nodulation [38,39].

Beneficial microbes produce a large number of MAMPs, such as flagellin and lipopolysaccharides (LPS), which can trigger host immunity [30]. Jacobs and associates demonstrated that PGPR could be recognized by the plant root immune system and triggered defense in a PTI-like manner at the early stage [40]. Leeman’s group [41] found that LPS, consisting of lipid A/innercore/O-antigen side chain, extracted from *P. fluorescens* WCS417 cell wall had the function of inducing systemic resistance against *Fusarium* wilt of radish. However, unlike pathogen-caused PTI, that often leads to severe cellular damage, beneficial microbes-induced immune responses were reported to be transient and relatively mild due to their host immune-manipulating mechanisms, performed in order to establish a mutually beneficial relationship with the host. It was shown that flg22 peptide, extracted from the beneficial *Burkholderia phytofirmans*, triggered only a small oxidative burst, which was enough to cause transient induction of defense genes without growth inhibition [32]. Furthermore, Millet and associates showed that the PGPR *P. fluorescens* WCS417 was able to suppress flagellin-triggered PTI responses in Arabidopsis roots via secretion of low molecular compounds [42]. Possibly, colonization of PGPR on the roots requires local suppression of PTI to protect the PGPR from MAMP-triggered antimicrobial compounds, which suggests a co-evolution leading to regulation of the host’s immune system after recognition of specific signals from beneficial microbes. Overall, these results demonstrated that beneficial microbes and their elicitors could induce plant defense responses, yet the mechanism of plant-specific recognition of beneficial microorganisms and immunity responses, that distinguish beneficial microorganisms from pathogens, is still unclear.

## 3. Early ISR Events Induced by Beneficial Microorganisms

Beneficial microorganisms are able to stimulate defense responses of host plants through different pathways, thereby endowing plants with resistance to multiple pathogens. *Bacillus amyloliquefaciens*, *B. atrophaeus*, *B. cereus*, *Pseudomonas fluorescens*, etc., were demonstrated to be effective against fungal, bacterial, and viral invasion through ISR (Table 1). Recent studies suggested that beneficial microbes induce early plant ISR events (Table 1), including, but not limited to, increased expression of pathogenesis-related PR genes, enhanced activities of defense-related substances, such as phenylalanine ammonia-lyase, polyphenol oxidase, peroxidase, β-1, 3 glucanase, and chitinase, and accumulating reactive oxygen species [43,44].

### 3.1. Reactive Oxygen Species

Under biotic or abiotic stress, plants produce a large number of reactive oxygen species (ROS), including superoxide anion (O^2−^), hydroxyl radical (OH), hydrogen peroxide (H_2_O_2_), and so on [71]. The induction of ROS is a significant signaling in control of various processes including immunity against pathogens, programmed cell death, and stomatal closure [72]. In Arabidopsis, the perception of MAMPs leads to a rapid, specific, and strong production of RBOHD-mediated ROS. RBOHD, a plant NADPH oxidase, is mainly controlled by Ca^2+^ via direct binding to EF hand motifs and phosphorylation by Ca^2+^-dependent protein kinases [73,74]. However, the accumulation of ROS also causes tissue cell damage [75]. Therefore, efficient scavenging of ROS by enzymatic and non-enzymatic reactions is necessary. Enzymatic ROS scavenging mechanisms in plants rely on peroxidase (POX), polyphenol oxidase (PPO), superoxide dismutase (SOD), ascorbate peroxidase (APX), glutathione peroxidase (GPX), and catalase (CAT), which are essential to the defense against ROS by reducing superoxide to H_2_O.

*B. cereus* AR156 activates plant defense response by inducing the accumulation of hydrogen peroxide and callose in plants and the activation of POD and SOD enzymes, mainly through SA and MAPK signaling pathways [76]. *Pseudomonas aeruginosa* 7NSK2 produced pyocyanin increases H_2_O_2_ in both local and distal leaves and induces resistance to blast disease (*Magnaporthe grisea*) but not sheath blight (*Rhizoctonia solani*). The opposite effect can be alleviated by co-application of pyocyanin and the antioxidant sodium ascorbate, suggesting that the reactive oxygen species can act as a double-edged sword in resistance against different diseases [56].

### 3.2. Callose Deposition

Callose is a β-1, 3 glucan polymer that accumulates in weak or compromised sections of plant cell walls under pathogen attack and plays an important role in plant sieve tube metabolism. Its synthesis and decomposition are directly related to the normal growth and metabolism of plants. Aniline blue staining was used to detect callose response to identify particular induced resistance-related genes involved in callose deposition. A study in 2009 illustrated the significance of PEN2 and PEN3 genes for pathogen resistance, required for callose deposition and consequently [77]. MAMPs released by PGPR generate ROS and increase the level of SA. High level of SA triggers callose deposition by regulating the PDLP5-dependent expression of callose synthase gene (*CALS10*) [78]. Endophytic bacterium *Pseudomonas fluorescens* strain 63–28 enhanced resistance to *Fusarium oxysporum* in tomato, through the rapid accumulation of callose and chitinases [79]. *Trichoderma harzianum* T-203 triggered plant systemic defense responses by increasing peroxidase and chitinase activities and forming barriers of callose [80].

### 3.3. Ca^2+^ Influx

Ion fluxes are immediately induced by elicitors, such as K^+^/H^+^ exchange, Cl^−^ effluxes, and Ca^2+^ influx, which play an important role in cell development and signal transportation, as well as in plant immunity [81]. Among these ion fluxes, Ca^2+^ influx is regarded as one of the most significant events, because of its role of a second messenger for many diverse physiological changes and cellular processes [81]. Some reports show that elicitor-induced Ca^2+^ influx not only mediates subsequent events, but also further amplifies Ca^2+^ signaling through Ca^2+^-dependent production of H_2_O_2_, which is able to increase Ca^2+^ influx from extracellular sources [82,83]. Pretreatment on bean (*Phaseolus vulgaris*) with forskolin, dibutyryl cAMP or Ca^2+^ ionophore A23187 enhanced the production of ROS to antagonize *Colletotrichum lindemuthianum*. In contrast, the Ca^2+^ channel blocker decreased the oxidative burst [84], suggesting that Ca^2+^ influx is required for ROS.

Calmodulin is a ubiquitous Ca^2+^ sensor, which can be activated by Ca^2+^ binding. Ca^2+^ and activated calmodulin further activate Ca^2+^/calmodulin-dependent protein kinase and protein phosphatase, membrane-bound enzymes, or transcription factors [85]. A large kinase family, known as Ca^2+^-dependent protein kinases (CDPK), with essential roles in plant defense responses, is regulated by binding of Ca^2+^. The application and colonization of PGPR, *Pseudomonas putida* MTCC 5279, activated calcium-dependent signaling by upregulating the expression of calcium-dependent protein kinase (*CPK32*) [86]. The Ca^2+^ signal can be non-linearly amplified upon binding of Ca^2+^, Ca^2+^ sensor relay proteins, calmodulin-binding transcription activators, and regulated transcription in plants [87]. Besides the functions on ROS, protein kinase cascades further the transfer of lipid signaling messengers and amplification of the elicitor signals to downstream reactions; another significant effect of Ca^2+^ spiking is differential activation of transcription factors, which directly regulate extensive defense gene expression [87,88,89]. A regulatory mechanism linking Ca^2+^ signaling to salicylic acid level is EDS1, an established regulator of salicylic acid level modulated by Ca^2+^/calmodulin-binding transcription factors [90]. The beneficial root-colonizing fungus *Mortierella hyalina* activated a Ca^2+^-dependent signaling pathway to resist *Alternaria brassicae* [64]. Cell wall extract of *Piriformospora* *indica*, a growth-promoting root endosymbiont, transiently alleviated cytosolic Ca^2+^ in Arabidopsis and tobacco through activating an important Ca^2+^ channel encoded by *CYCLIC NUCLEOTIDE GATED CHANNEL 19* (*CNGC19*) in the mutualistic interaction between beneficial microbe and plant [91,92].

### 3.4. Transcriptional Factors

Several crucial transcriptional factors are involved in the regulation network of ISR through JA or/and ET signaling pathway. WRKY transcription factors are implicated in the responses to plant–microbes interactions. The Arabidopsis thaliana WRKY genes are differentially expressed in a time-dependent manner during the plant interaction with beneficial fungus *T. atroviride*. The expression of positive regulators in JA-mediated pathway, such as AtWRKY8 and AtWRKY33, was more anticipated than the expression of the WRKY genes regulated by SA pathway [67]. WRKY11 and WRKY70 were involved in the regulation of *B. cereus* strain AR156-triggered ISR in Arabidopsis, through the JA and SA signaling pathways, respectively [93]. MYB family proteins function as transcriptional factors regulating plants development and responses to biotic and abiotic stress [94]. MYB72 was activated upon colonization of *P. fluorescens* WCS417r and was required in the early signaling steps of beneficial microbe-mediated ISR by acting upstream of ethylene in the signaling pathway [95]. The basic helix-loop-helix (bHLH) transcription factor MYC2 was required for beneficial microbe-triggered ISR, while its function was targeted by pathogens through effector-mediated suppression of host immunity [96]. Ethylene response factor1 (ERF1) encodes a transcription factor that regulates the expression of pathogen response genes that prevent disease progression. The expression of ERF1 can be activated rapidly and synergistically by both JA and ET [97]. There are two branches, the MYC branch and the ERF branch, in the JA signaling pathway responding to wounding stress and necrotrophic pathogen attack, regulated by MYC-type transcriptional regulator and APETALA2/ethylene response factor (AP2/ERF) family of transcriptional regulator, such as ERF1 and ORA59, respectively [98]. Future attempts to unravel more detailed regulatory mechanisms on transcription factors involved in beneficial microorganism-mediated ISR will improve our understanding of the formation and regulation of ISR.

### 3.5. Defense-Related Genes

Defense mechanisms of ISR depend on an accurate and context-specific regulation of gene expression. Interactions between genes and their products result in complex circuits and form a regulatory network. Timmermann et al. explored the regulatory mechanism of the ISR defense response triggered by the beneficial bacterium *Paraburkholderia phytofirmans* PsJN and drew a regulatory network according to gene expression and time series data [99]. The Plant Defensin 1.2 (encoded by PDF1.2; AT5G44420) has previously been proved to accumulate systemically via a SA-independent pathway in leaves of Arabidopsis upon challenge by fungal pathogens and play a role as a marker of the JA signaling pathway [100,101]. As previously mentioned, some SA-dependent PR genes express antimicrobial proteins. Notably, the activation of PR1, PR2, and PR5 depend on SA signaling, whereas PDF1.2, as well as PR3 and PR4 genes, are activated via an SA-independent and JA-dependent pathway [102]. Although it was proposed that PR genes were irrelevant with ISR after certain beneficial microbe treatment [19,103], pretreatment with non-pathogenic *B. cereus* AR156 triggered expression of PR1, PR2, PR5, and PDF1.2 of *Arabidopsis thaliana*, which indicated the activation of SA and JA/ET signaling pathways, respectively [21,104,105]. The loss-function mutant of NPR1, an important regulatory factor in the SA-dependent pathway [106,107], was able to express neither ISR nor SAR [103]. Based on the previous research results, NPR1 coordinates SA and JA signaling pathway and regulate downstream defense response genes [108].

### 3.6. Secondary Metabolites

Under natural conditions, plants produce a vast array of secondary metabolites, which are critical for plant adaptation to abiotic and/or biotic stresses. Plant secondary metabolites are able to interact with beneficial microbes and modulate plant growth and immune process, and inhibit growth or metabolism of pathogenic microorganisms. PGPR can be recruited by root exudates, which structure a special community of rhizosphere microorganisms and enhance biofilm formation of beneficial microbes [109]. Biochemical evidence showed that plant roots secreted L-malic acid (L-MA) to selectively recruit beneficial rhizobacteria, such as *B. subtilis* FB17 [110]. Metabolites derived from the tryptophan and phenylpropanoid pathways, such as flavonoids, play roles in plant interactions with beneficial and pathogenic microbes, and these pathways are regulated by nutrient availability [111]. The relative abundance of root-associated *Acidobacteria*, *Gaiellales*, *Nocardioidaceae*, and *Thermomonosporaceae* in the soil can be affected by the flavonoid (7,40-dihydroxyflavone) excreted from *Medicago sativa* [112]. Moreover, the flavonoids, such as luteolin, from the leguminous plants can act as growth regulators as well as signaling molecules for *Rhizobium* bacteria to initiate symbiosis [113]. Plants also release strigolactones that stimulate the branching of hyphae of arbuscular mycorrhizal fungi to establish beneficial symbiosis [114]. Camalexin and glucosinolates are required for the *P. fluorescens* SS101-induced SA signaling-dependent resistance against *Pst* [58].

In turn, the secondary metabolites secreted by beneficial microorganisms can directly antagonize pathogenic bacteria and act as immune elicitors to raise ISR [115]. Phenazines produced by beneficial *Pseudomonas* bacteria showed antifungal activity and were able to elicit ISR [116]. *B. cereus* AR156 extracellular polysaccharides (EPS) could induce systemic resistance to *Pst* DC3000 in *Arabidopsis* [76]. Lipopolysaccharides (LPS), as MAMP molecules, triggered the activation of signal transduction pathways involved in phytohormones SA and JA, and the associated methyl esters and sugar conjugates [117]. Harzianic acid produced by *Trichoderma harzianum* M10-induced, modulated signaling pathway and differentially expressed genes (DEGs) involving JA/ET- and SA-mediated signaling pathways, and increased reactive oxygen species (ROS) [69]. Microbial volatile compounds (MVCs) have been shown to promote plant growth via improved photosynthesis rates, enhanced immune system, and activated phytohormone signaling pathways [118]. Critical reviews have shown the effects of VOCs on ISR and their interactions with SA, JA/ET, and auxin signaling pathways [119,120,121]. Cyclic lipopeptides surfactin and VOC 2,3-butanediol, produced by Bacillus spp., have been identified as elicitors of ISR [46,122]. These results illustrate the network of interaction between plant and beneficial microorganisms, in which plants generate metabolites to recruit beneficial microbes and inhibit harmful microbes, and beneficial microbes secrete secondary metabolites to enhance resistance of host plants.

### 3.7. Stomatal Regulation

Stomata play an important role in plant photosynthesis, respiration, and transpiration. Although stomatal closure decreases gas exchange, resulting in the reduction in photosynthetic activity, this reaction is actually a part of a plant innate immune response to restrict bacterial invasion [123]. Abscisic acid (ABA) plays significant roles in the regulation of stomatal aperture. ABA is produced under stress. The cellular ABA receptors bind to ABA and interact with a group of type 2C protein phosphatases (PP2C) [124,125], inactivating the inhibitory regulatory function of PP2C, but activating SnRK2 protein kinase OST1 [126]. Activated OST1 binds directly to and phosphorylates the anion channel slow anion channel-associated1 (SLAC1), mediating anion release from the guard cells and promoting stomatal closure [127,128,129]. ROS play a key role in ABA-controlled, hyperpolarization-activated Ca^2+^ channels in the plasma membrane of guard cells [130]. The production of H_2_O_2_ can be catalyzed by OST1 [131,132]. Lipoxygenase encoding gene LOX1, also known as a JA-responsive gene, is expressed in guard cells in response to PAMPs and is required to trigger stomatal defense [133], indicating the JA signaling pathway participates in the regulation of stomatal defense. PGPR *B. amyloliquefaciens* FZB42 mediates ABA and JA pathways and produce acetoin and 2,3-butanediol to induce stomatal closure in response to biotic stress [45,134,135], which suggests multiple signaling components coordinate in stomatal regulation.

## 4. Induced Signaling Transduction Pathway

Systemic acquired resistance (SAR) is generally considered to be induced by pathogenic microbes, while induced systemic resistance (ISR) is caused by beneficial microbes. SAR often results in increasing level of SA and coordinating activation of pathogenesis-related (PR) genes, such as PR1, PR2, and PR5, and involves one or more long-distance signals that transduce an enhanced immune signal to undamaged plant parts [136]. ISR is commonly regarded as SA-independent and develops without accumulation of PR proteins [19]; however, there are a few exceptions where identified ISR occurs in an SA-dependent manner. For example, *Pseudomonas aeruginosa* 7NSK2 induce systemic resistance with higher innate SA accumulation and PAL activity by producing nanogram amounts of SA [137]. ISR is identified to be activated through the JA/ET-dependent signaling pathway, involving *plant defensin 1.2* (*PDF1*.*2*) [101]. Our previous results showed that pretreatment with non-pathogenic *B. cereus* AR156 triggered expression of *PR* genes and *PDF1.2* of *Arabidopsis thaliana*, which indicated that SAR and ISR were stimulated in SA and JA/ET signaling pathways, respectively [21]. It was demonstrated that simultaneous activation of SAR and ISR pathways resulted in an additive effect in an NPR1-dependent manner against *Pseudomonas syringae* pv. *tomato* (*Pst*) [138]. It is difficult to distinguish SAR and ISR, both of which activate the pathogenic related genes and increase the accumulation of reactive oxygen species (ROS) and callose. SAR stimulates a rapid response to pathogens, and this signal can be conferred in a short time. In contrast, plants activated by beneficial microbes are in a special ISR state called “priming”, ready to give faster and stronger defense responses.

Jasmonates (JAs) are fatty acid-derived signaling components involved in the regulation of development and defense response in plant [9]. It was reported that beneficial microbe-mediated ISR is JA/ET-dependent by enhancing sensitivity to hormones rather than enhancing the production level or expression of JA/ET-responsive genes [139]. In addition, activation of JA signaling by application of methyl jasmonate (MeJA) not only regulates the level of resistance, but also influences structure of rhizosphere microbial community, including the species known to suppress plant disease [140]. Salicylic acid (SA) has been shown to be a required signal molecule in SAR. SA level increased after microbes infection, and SA acts as an endogenous signal with rapid movement in phloem that triggers accumulation of PR proteins [141]. SA biosynthesis seems under direct control of *SID2* and *EDS5* genes, while the *EDS1*, *EDS4*, and *PAD4* genes play regulation functions in the synthesis of SA [142,143]. It was reported that the Arabidopsis mutants *enhanced disease susceptibility1* (*eds1*), *eds4*, *eds5*, *phytoalexin deficient4* (*pad4*) and SA *induction deficient2* (*sid2*) failed to accumulate SA and were more susceptible to *P. syringae* [144]. It is generally believed that salicylic acid (SA) signaling is linked with plant resistance against biotrophic and hemibiotrophic pathogens, while jasmonic acid (JA)/ethylene (ET) signaling provokes host resistance to necrotrophic pathogens [137,145]. Although the initial research disregarded the involvement of SA in beneficial microbe-induced systemic resistance, recent studies have shown that beneficial microorganisms can control plant disease through activating SA and JA/ET signaling pathways. Beneficial microbes, such as *Bacillus* and *Trichoderma*, showed the ability to increase the expression of SA and JA/ET marker genes *PR1* and *LOX2*, respectively, and increased the content of SA and JA in plants [22,23,24].

Phytohormone crosstalk is crucial for plant defense against pathogens and insects. Crosstalk between SA- and JA-dependent pathways are generally considered to be antagonistic [146]. SA synthesis-deficient Arabidopsis plants produced 25-fold higher levels of JA and enhanced expression of the JA-responsive genes *LOX2*, *PDF1.2* in response to infection by *P. syringae* DC3000 [106]. Mitogen-activated protein kinases (MAPKs) and their cascades were shown to transduce various extracellular stimuli into internal cellular responses. MPK3 and MPK6 are positive regulators of plant defense responses controlling JA and ET biosynthesis [138,147]. MAPKs are required in JA biosynthesis and ET production [148,149,150] and participate in the regulation of the ROS burst [151]—which, on the contrary, negatively regulates SA-induced defense responses [82]. However, recent studies also revealed the synergistic interactions of SA and JA/ET signaling pathways in beneficial microbe-induced systemic resistance. Simultaneous activation of SAR and ISR pathway resulted in an additive effect in a NPR1-dependent manner against *P. syringae* pv. *tomato* (*Pst*) [152]. *B. cereus* AR156 was able to activate SA- and JA/ET-dependent signaling pathways simultaneously [21], and rapidly activate MAPK signaling and FRK1/WRKY53 gene expression by leaf infiltration [104].

## 5. Regulatory Role of Small RNAs

Small RNAs, including microRNAs (miRNAs), small interfering RNAs (siRNAs), and piwi-interacting RNAs (piRNAs), are noncoding RNAs with approx. length of 20–30 nucleotides, with important roles in regulating biological processes, such as development, reproduction, and stress responses [153]—collectively termed RNA interference (RNAi). Small RNAs are generated by DICER or DICER-like (DCL) proteins and then loaded into RNAi effector proteins Argonautes (AGOs) for regulating the expression of target mRNA through transcription or translation inhibition [154]. Plant miRNA precursors, possessing imperfectly base-paired hairpin loop structures, are first transcribed by RNA polymerase II and then cut by DCL endonuclease to produce miRNA/miRNA* double-stranded RNA. The double strand consists of a guide strand (mature miRNA) and a passenger strand (miRNA*), one of which binds to AGOs to form an active RNA induced silencing complex (RISC) [155]. In contrast to miRNAs, siRNAs are derived from perfectly paired double-stranded RNA (dsRNA) precursors. These dsRNA precursors are derived either from antisense transcription or by the action of a cellular RNA-dependent RNA polymerase (RDR) [154].

It is evident that small RNAs play crucial roles in plant innate immunity against virus, bacteria, and fungi [135,156,157]. The first miRNA identified to involve in PTI is miR393, which is induced by flg22 to repress auxin signaling by silencing its receptors [158]. Emerging evidence indicates that plants microRNAs target conserved domains of NB-LRR-encoding genes and trigger ETI [159]. *B. cereus* AR156 pretreatment triggers ISR signaling and downregulates the miR825/miR825* pair, which targets toll-interleukin-like receptor NB-LRR (TNLs)-type resistance (R) genes [48,49]. In addition, *B. cereus* AR156 triggers ISR against *P. syringae* pv. *tomato* DC3000 by suppressing miR472 and activating coiled-coil NB-LRR-mediated basal immunity in Arabidopsis [160]. *B. amyloliquefaciens* FZB42 inoculation suppresses Arabidopsis specific miR846 expression to induce systemic resistance via a JA-dependent signaling pathway [135]. A total of 146 known miRNAs and 217 novel miRNAs were identified to be differentially expressed in maize in response to FZB42 and loss-of-function mutant FZB42 Δ*sfp* Δ*alss* (deficient in triggering ISR). Among those, four miRNAs (zma-miR169a-5p, zma-miR169c-5p, zma-miR169i-5p, and zma-miR395b-5p), specifically depressed in FZB42 treatment, were selected as candidates of ISR-associated miRNAs [161].

Small RNAs play a significant role in RNA silencing in universal eukaryotic gene expression regulation. Small RNA [162] and its function of mediating RNAi were first reported in *Caenorhabditis elegans* [163]. It has been shown that small RNAs can spread among different organisms and induce gene silencing of each other, which is also known as cross-kingdom RNAi [164]. Small RNAs from pathogens and pests move into the host plant to inhibit plant immunity; in turn, host-delivered RNA interference plays an important role in regulating host immunity against bacteria, fungi, oomycetes, viruses, and pests. Cross-kingdom, post-transcriptional gene silencing can also occur between the symbiotic organisms, such as AMF and the host plant, during the regulation of symbiosis [165,166]. Based on the naturally occurring cross-kingdom RNAi between the beneficial microorganisms/pathogens–plants, it is possible to achieve host-induced gene silencing (HIGS) by transgenic expression of genes encoding pathogenic double-stranded RNA (dsRNA) in the host to control plant diseases [167]. In addition, in vitro synthesized dsRNA can be directly sprayed to and absorbed by host plants or harvested fruits, circumventing the transgenic risk, and resulting in gene silencing of target pathogen/insect pests (called spray induced gene silencing, SIGS) [168,169]. The intrinsic advantages of HIGS and SIGS offer them the potential to develop new strategies for crop disease management.

## 6. Conclusions and Discussion

**Figure 1:**
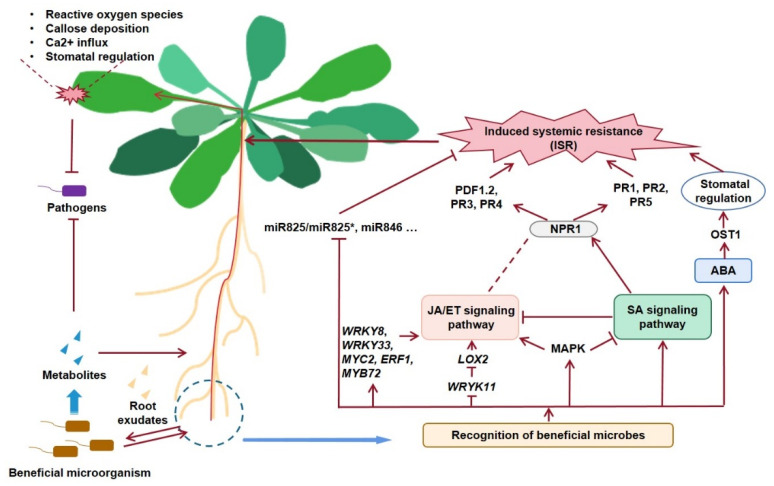
Working model of beneficial microorganism-mediated ISRIn the next stage, there are still some problems to be solved. More microbial germplasm resources with biocontrol potential remains to be discovered; the formulation and shelf life of bacteria need to be improved; mining and identification of new antibacterial substances and analysis of their biosynthesis pathway, research on the genetic regulatory network of biosynthesis and microbial metabolites, and its application, based on genetic modification, are also interesting topics. Efficient and stable RNAi technology requires mastering the proper design and synthesis of dsRNA. The screening carriers of dsRNA are also indispensable to develop and improve the application of RNAi technology in plant disease control.

In this review, we discussed the recognition mechanisms of the plant to beneficial microbes. Beneficial microbes can be recognized as MAMPs by PRR and stimulate the host plant immune response. In order to build symbiosis relationship with the plant host, beneficial microbes evolved to be able to minimize stimulation of their host’s immune system. However, there is still an urgent need for detailed research about the mechanism on the balance between efficient recognition and strength of host immune response. The genes and transcriptional factors participating in defense response make up a complicated network through the signaling crosstalk. As mentioned, SA and JA can be activated by beneficial microorganisms at the same time in an NPR1-dependent pathway. In addition, SA, JA, ET, and MAPK cascades interact with each other, and coordinate in the downstream defense response. Moreover, non-coding RNAs, induced by beneficial microorganisms, play a vital role in regulating the host development and resistance to the pathogen (Figure 1). Therefore, genome-wide profiling of miRNA and the subsequent functional verification are two important projects to explore in the future, and RNA interference technology can be a sound method to control plant diseases and pests.

## Figures and Tables

**Table 1:** Resistant mechanism of beneficial microbes.

Strains	Pathogens	Diseases	Main Resistance Mechanisms	References
*Bacillus amyloliquefaciens* Ba13	Tomato yellow leaf curl virus	Tomato yellow leaf curl virus disease	*PR1*, *PR2*, and *PR3* gene (antimicrobial effects, beta-1,3 glucanase, and chitinase activities); enhanced activities of phenylalanine ammonia lyase (regulation of plant growth and stress tolerance), beta-1,3 glucanase (inhibition of the mycelial growth and spore germination), and chitinase (inhibition of mycelial growth).	[43]
*Bacillus amyloliquefaciens* FZB42	*Phytophthora nicotianae*, *Rhizoctonia solani*	Leaf disease, bottom rot	ABA/SA (FZB42-induced stomatal closure); stomatal closure (reduction in pathogen invasion); defense-related genes- PR-la, LOX, and ERF1 (defense effects); secondary metabolites—surfactin, fengycin, and bacillomycin D (direct antagonistic effect and induction of defense-related genes).	[45,46]
*Bacillus atrophaeus* GBSC56	*Meloidogyne incognita*	Root-knot nematode	Volatiles-dimethyl disulfide, methyl isovalerate, and 2-undecanone (regulation of antioxidant enzymes, protection from oxidative stress, and against *M. incognita*).	[47]
*Bacillus cereus* AR156	*Pseudomonas syringae* pv. tomato (Pst) DC3000		Suppression of miR825 and miR825* (activating the targeted defense-related genes).	[48,49]
*Bacillus cereus* C1L	*Botrytis cinerea*, *Cochliobolus heterostrophus*	Foliar and soil diseases	Volatile metabolites-dimethyl disulfide (induction of ISR).	[50]
*Bacillus megaterium* DE BABY TRS-4	*Fomes lamaoensis*	Brown root rot	Enzymes activity-peroxidase, chitinase, beta-1,3-glucanase (inhibition of the mycelial growth and spore germination), and phenyl alanine ammonia lyase (regulation of plant growth and stress tolerance); enhanced phosphate solubilization and production of IAA (promotion of plant growth); regulation of siderophore and antifungal metabolite (inhibition of pathogen growth).	[51]
*Bacillus subtilis* FB17	*Pseudomonas syringae* pv. tomato (Pst) DC3000		Malate efflux (enabling stable colonization).	[52]
*Bacillus subtilis* M4	*Colletotrichum lagenarium*, *Pythium aphanidermatum*		Metabolic and transcriptomic changes (enhanced defense response).	[27]
*Bacillus subtilis* OTPB1	*Alternaria solani*, *Phytophthora infestans*	Early and late blight	Defense-related enzymes—peroxidase, polyphenol oxidase, and superoxide dismutase (inhibition of the mycelial growth and spore germination, and protection from oxidative stress).	[53]
*Bacillus subtilis* UMAF6639	*Podosphaera fusca*	Cucurbit powdery mildew	Reactive oxygen species (inhibition of the mycelial growth and spore germination); cell wall reinforcement (reduction in pathogen invasion); metabolites—surfactin lipopeptide (stimulation of the immune response).	[54]
*Paenibacillus alvei* K165	*Verticillium dahliae*		*PR-1*, *PR2*, and *PR-5* genes (antimicrobial effects, beta-1,3 glucanase, and chitinase activities, markers for SA-mediated activation of SAR).	[55]
*Pseudomonas aeruginosa* 7NSK2	*Magnaporthe grisea*; *Rhizoctonia solani, Botrytis cinerea*	Rice blast and sheath blight	Metabolites-phenazine pyocyanin and pyochelin (induction of ISR); ROS (inhibition of the mycelial growth and spore germination); SA (expression of acquired resistance).	[56,57]
*Pseudomonas fluorescens* SS101	*Pseudomonas syringae* pv tomato (Pst)		Metabolic and transcriptomic changes (induction of resistance responses).	[58]
*Pseudomonas fluorescens* PTA-CT2	*Plasmopara viticola, Botrytis cinerea*	Downy mildew and gray mold diseases	Activation of SA, JA, and ABA defensive pathways, HR (reduction in pathogen invasion).	[59]
*Pseudomonas fluorescens* WCS417		Broad spectrum	Transcription factor MYB72 (regulation of iron-uptake responses).	[60]
*Streptomyces lydicus* M01	*Alternaria alternataon* cucumbers	Foliar disease	ROS (inhibition of the mycelial growth and spore germination).	[61]
*Streptomyces pactum*	Tomato yellow leaf curl virus	Tomato yellow leaf curl virus disease	ROS (inhibition of the mycelial growth and spore germination); enzyme activity-peroxidase, chitinase, beta-1,3-glucanase (inhibition of the mycelial growth and spore germination), and phenyl alanine ammonia lyase (regulation of plant growth and stress tolerance); defense-related genes *PR-1*, *PR2*, and *PR-5* genes (antimicrobial effects, beta-1,3 glucanase, and chitinase activities, markers for SA-mediated activation of SAR); JA/ET (induction of immune response and reduction in pathogen invasion).	[62]
*Acrophialophora jodhpurensis*	*Rhizoctonia solani* AG4-HG II	Tomato root and crown rot	Direct antagonistic activity; ROS (inhibition of the mycelial growth and spore germination); enzyme activity—peroxidase, chitinase, beta-1,3-glucanase (inhibition of the mycelial growth and spore germination), and phenyl alanine ammonia lyase (regulation of plant growth and stress tolerance); iron restriction (inhibition of pathogen growth and promotion of plant growth).	[63]
*Mortierella hyalina*	*Alternaria brassicae*		JA (response to external and biological stresses); Ca^2+^ (regulating the permeability of plant cell membrane, enhance resistance).	[64]
*Serendipita vermifera*	*Bipolaris sorokiniana*		ROS (inhibition of the mycelial growth and spore germination); enzyme activity—hydrolytic enzymes (activation of defence).	[65]
*Trichoderma atroviride*	*Botrytis cinerea*		Glutamate: glyoxylate aminotransferase GGAT1 (stimulation of plant growth and induction of the plant systemic resistance); WRKY transcription factors (active defense response to biotics and abiotic stresses).	[66,67]
*Trichoderma harzianum*	*Bipolaris sorokiniana*, *Rhizoctonia solani*	Spot blotch, wilt	Phenylpropanoid activities (reduction in cell wall disruption and tissue disintegration and increased suberization and lignification of the plant cell); secondary metabolite Harzianic acid (inducing the expression of several genes involved in defense response).	[68,69]
*Trichoderma longibrachiatum* MK1	*Botrytis cinerea*, *Alternaria alternata, Pythium ultimum*, and *Rhizoctonia solani*		Type II hydrophobin (direct antifungal as well as a microbe-associated molecular pattern and a plant growth promotion (PGP) activity).	[70]
*Trichoderma harzianum* OTPB3	*Alternaria solani*, *Phytophthora infestans*	Early and late blight	Defense-related enzymes—peroxidase, polyphenol oxidase, and superoxide dismutase (inhibite the mycelial growth and spore germination, and protection from oxidative stress).	[55]

## Data Availability

Not applicable.
